# Dentin dysplasia type I: a case report and review of the literature

**DOI:** 10.1186/1752-1947-4-1

**Published:** 2010-01-07

**Authors:** Lida Toomarian, Fatemeh Mashhadiabbas, Mahkameh Mirkarimi, Leili Mehrdad

**Affiliations:** 1 Pediatric Department, Shahid Beheshti University, Tehran, Iran; 2 Oral and Maxillofacial Pathology Department, Shahid Beheshti University, Tehran, Iran; 3 Pediatric Department, Zahedan University of Medical Sciences, Zahedan, Iran; 4 Private practice, Tehran, Iran

## Abstract

**Introduction:**

Dentin dysplasia is a rare hereditary disturbance of dentin formation characterized by defective dentin development with clinically normal appearing crowns, severe hypermobility of teeth and spontaneous dental abscesses or cysts. Radiographic analysis shows obliteration of all pulp chambers, short, blunted and malformed or absent roots and peri-apical radiolucencies of non carious teeth.

**Case presentation:**

We present a case of dentin dysplasia type I in a 12-year-old Iranian boy, and the clinical, radiographic and histopathologic findings of this condition and treatment are described.

**Conclusions:**

There are still many inconclusive issues in the diagnosis and management of patients with dentin dysplasia. The diagnostic features of this rare disturbance will remain incompletely defined until additional cases have been described. Early diagnosis of the condition and initiation of effective regular dental treatments may help these patients to prevent or delay loss of dentition.

## Introduction

Dentin dysplasia (DD) is an autosomal dominant hereditary disturbance in dentin formation, which may present with either mobile teeth or pain associated with spontaneous dental abscesses or cysts. It is a rare anomaly of unknown etiology that affects approximately one patient in every 100,000 [1]. The condition was first described by Ballschmiede [2] but it was Rushton [3] who termed the condition dentinal dysplasia. This condition is rarely encountered in dental practice. In 1972, Witkop [4] classified DD into two types, radicular DD as type I and coronal DD as type II. In type I, both the deciduous and permanent dentitions are affected. The crowns of the teeth appear clinically normal in morphology but defects in dentin formation and pulp obliteration are present. Radiographic examination is important for the identification of DD type I. There are four subtypes for this abnormality. In type 1a, there is no pulp chamber and root formation, and there are frequent periradicular radiolucencies; type 1b has a single small horizontally oriented and crescent shaped pulp, and roots are only a few millimeters in length and there are frequent peri-apical radiolucencies; in type 1c, there are two horizontal or vertical and crescent shaped pulpal remnants surrounding a central island of dentine and with significant but shortened root length and variable peri-apical radiolucencies; in type 1d, there is a visible pulp chamber and canal with near-normal root length, and large pulp stones that are located in the coronal portion of the canal and create a localized bulging in the canal, as well as root constriction of the pulp canal apical to the stone and few peri-apical radiolucencies [5]. Histologically, the enamel and the immediately subjacent dentin appear normal. Deeper layers of dentin show an atypical tubular pattern with an amorphous, atubular area and irregular organization. Pulpally to normal appearing mantle dentin, and globular or nodular masses of abnormal dentin are seen [6]. It is not known if DD type I is another allelic disorder of the dentin sialophosphoprotein (DSPP) gene, or a mixed phenotype [1].

This article describes an uncommon case of DD type I, subtype 1a, in a 12-year-old Iranian boy, highlighting the clinical and radiographic variations of the defect as confirmed by histopathologic examination.

## Case presentation

A 12-year-old Iranian boy was referred to the Pedodontics Department of Shaheed Beheshti University Medical Sciences, Dental School due to excessive painful swelling of his mandibular left cheek region. Clinical examination showed that the following teeth were present in his mouth:

7654321 12 C4567

7654321 12 C4567

**Figure 1:**
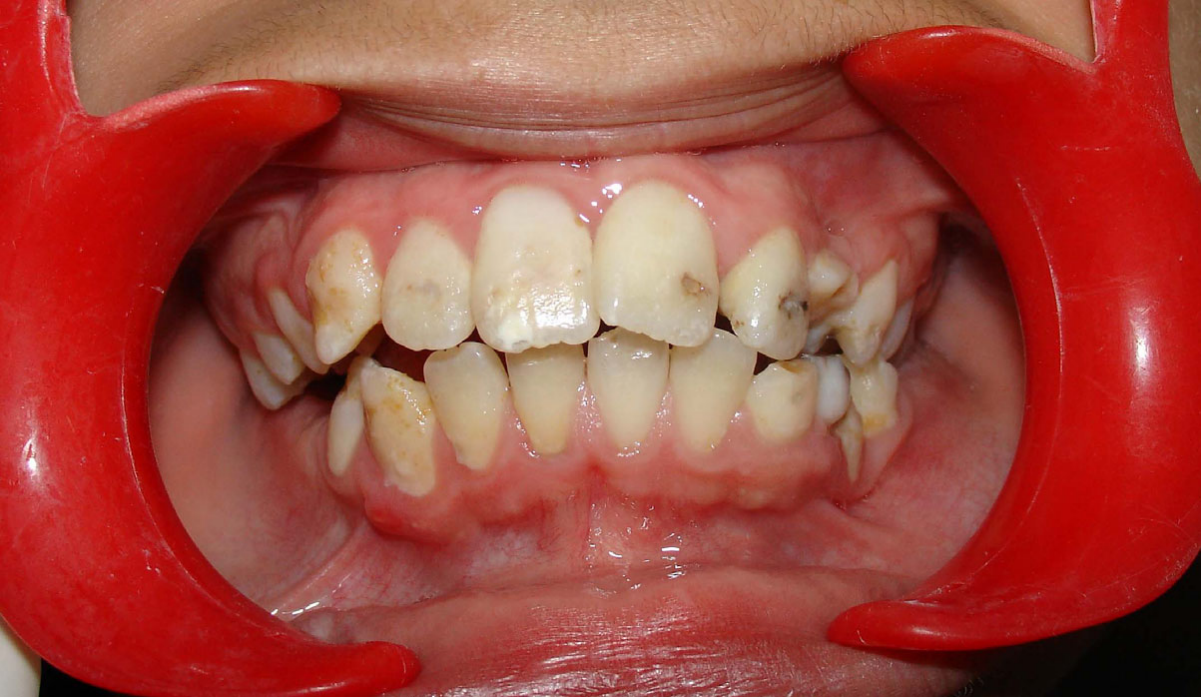
**Intra-oral image before treatment**.

**Figure 2:**
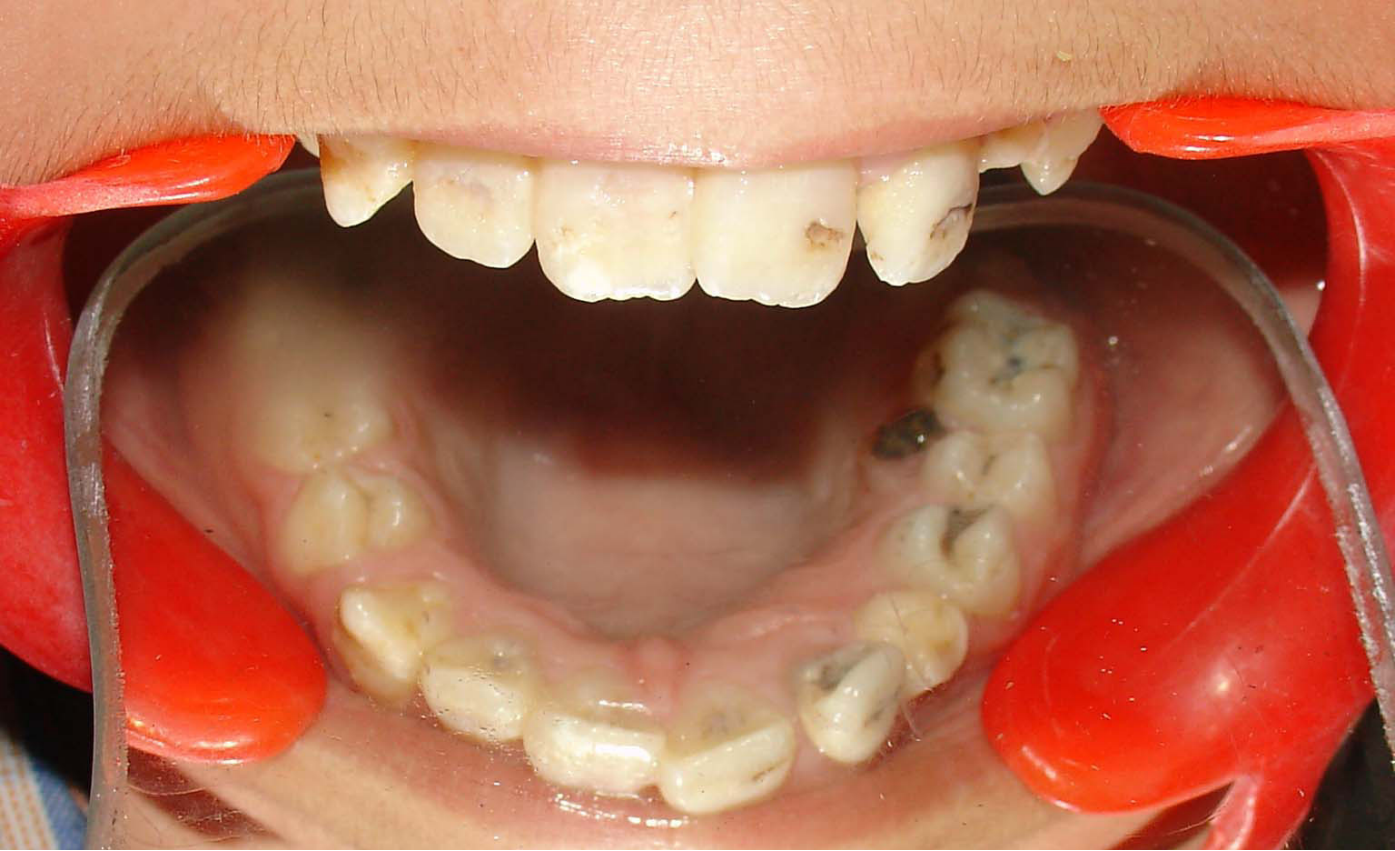
**View of maxillary teeth before treatment**.

**Figure 3:**
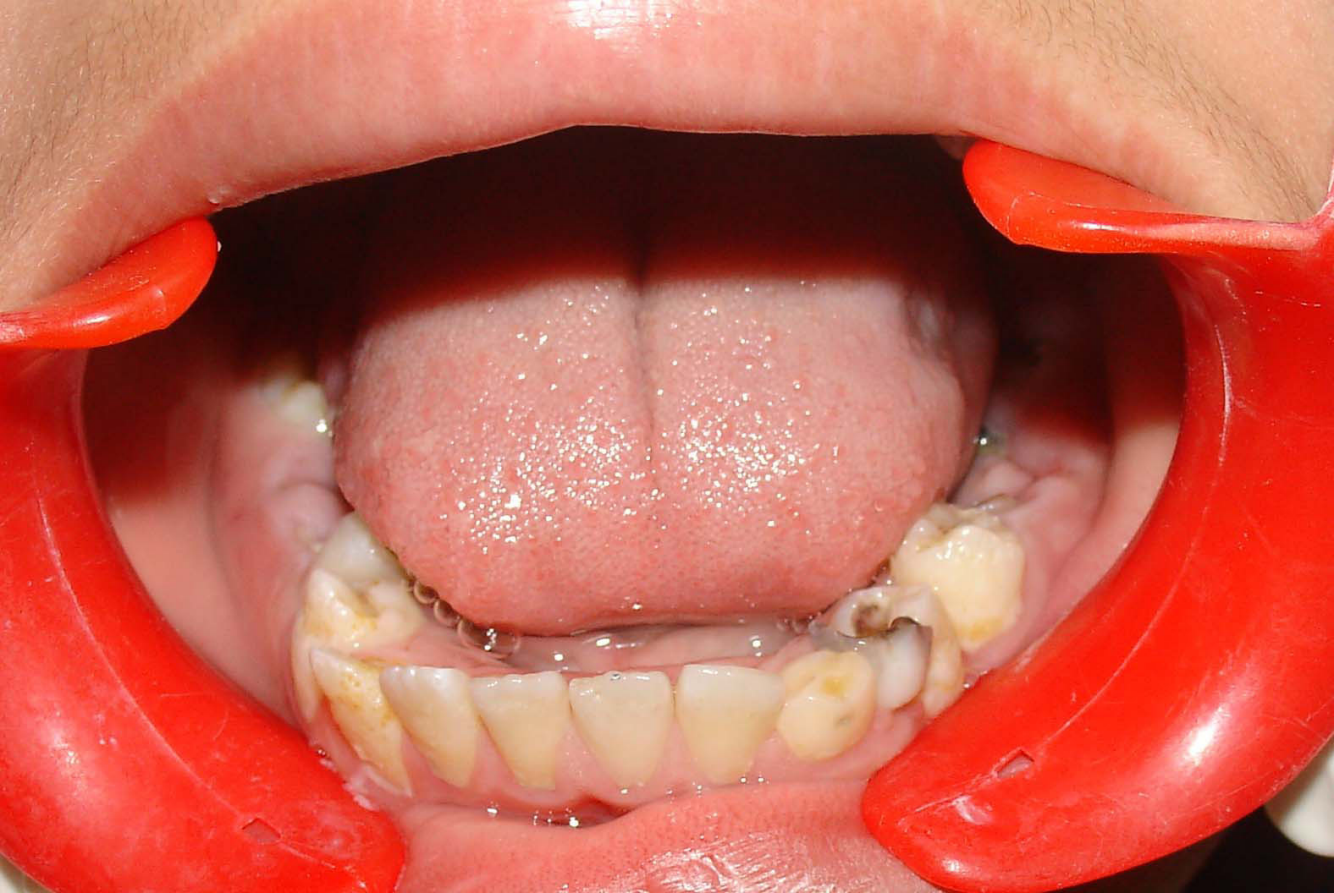
**View of mandibular teeth before treatment**.

**Figure 4:**
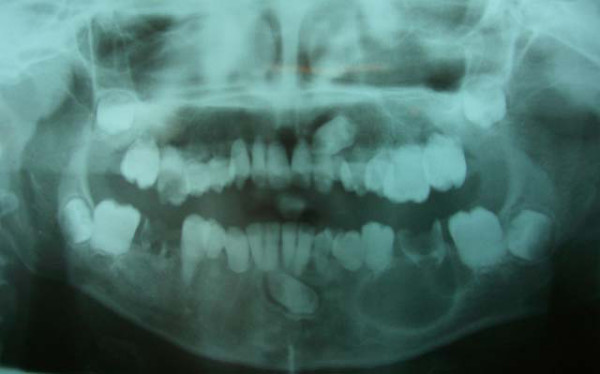
**Panoramic radiography before treatment**.

The crowns of his teeth had normal morphologic characteristics, but the color of his teeth was slightly more yellow than expected for a patient of his age (Figure 1, Figure 2, Figure 3). Oral hygiene was poor and there were plaque deposits present in all quadrants. The patient's medical history revealed no evidence of disturbance in general health. Caries were present in most of the teeth. The maxillary and mandibular central and lateral incisors were mobile, and there was a painful expansion on the buccal region of the mandibular left first molar. Information supplied by his mother indicated that the child's gingiva had become markedly swollen in both upper and lower jaws on various occasions and that this condition had been treated by antibiotic therapy. Radiographic examination revealed pulpless teeth with no root formation in most teeth and roots of only a few millimeters in some teeth. There was a well-defined round unilocular radiolucency in association with the apex of the left first permanent molar. The left maxillary and mandibular canine teeth were impacted and located horizontally in panoramic view (Figure 4).

On the basis of the clinical and radiographic appearance, a diagnosis of DD type I, subtype 1a, was suspected. Clinical and radiographical examination of the patient's parents and siblings revealed no cases of DD type I, and there were no previous cases of this disturbance in the familial history. The following treatment plan was formulated: dietary and oral hygiene instructions, fluoride supplements, surgical enucleating of the cystic lesion at the left first permanent molar region, restoration of the carious teeth, and extraction of the left primary canine and primary first molar, which were carious and mobile.

**Figure 5:**
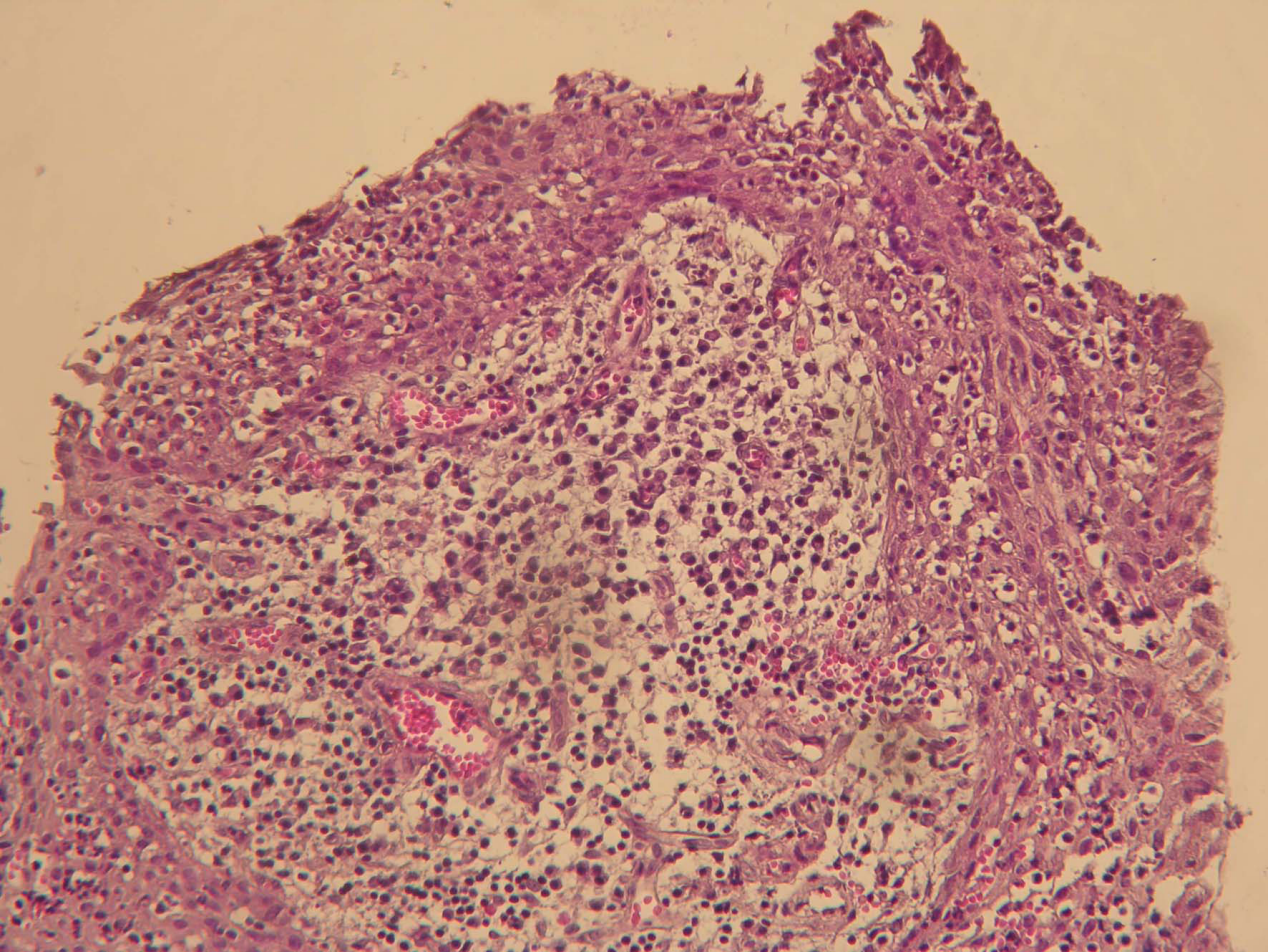
**In histopathologic examination, a variable thickness of non-keratinized stratified squamous epithelium with arch-shaped appearance is evident (×40)**.

**Figure 6:**
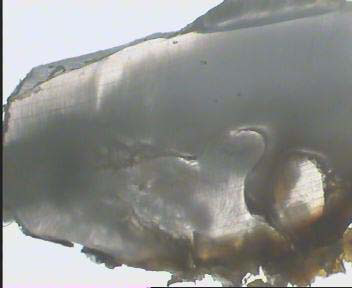
**Ground section view with stereomicroscope showing normal coronal dentin and irregular dentine obliterating the pulp chamber**.

The cystic lesion was enucleated and sent for histopathological examination. Based on the pedodontist's and surgeon's view, it was decided to extract the remaining root of the lower left first permanent molar during surgery because of extensive caries. The histopathological features were consistent with the clinical diagnosis of a radicular cyst. The cystic cavity was lined with a variable thickness of non keratinized stratified squamous epithelium with arch-shaped appearance and exocytosis in the underlying connective tissue was severely infiltrated by chronic inflammatory cells. Extravasated red blood cells and hemosiderin pigments were also seen (Figure 5). The extracted primary teeth were sent for histological examination. The ground section was examined with a stereomicroscope: the superficial dentin of the crown appeared normal, but the pulp chamber was obliterated by an unusual type of calcified material consisting of dentin, and deeper layers of dentin had an atypical tubular pattern (Figure 6). These features are consistent with those of DD type I, confirming the diagnosis based on the clinical and radiographic features. It is anticipated that more permanent teeth may be lost due to severe mobility and may undergo spontaneous pulpal necrosis. The possibility of endosseous implants is being explored for when the patient reaches his late teens and growth is complete.

## Discussion

The pathogenesis of DD is still unknown in the dental literature. Logan *et al*. [7] proposed that it is the dentinal papilla that is responsible for the abnormalities in root development. They suggested that multiple degenerative foci within the papilla become calcified, leading to reduced growth and final obliteration of the pulp space. Wesley *et al*. [8] proposed that the condition is caused by an abnormal interaction of odontoblasts with ameloblasts leading to abnormal differentiation and/or function of these odontoblasts. Dentin dysplasia type I should be differentiated from dentin dysplasia type II, dentinogenesis imperfecta and odontodysplasia. In our patient, the calcified pulp chambers, rootless teeth, peri-apical radiolucent areas and the nature of the peri-apical lesion are characteristic findings for the diagnosis of DD type 1, sub type 1a. DD is usually an autosomal dominant condition [1], but in this patient, there was no familial history of the disease, so he is considered to be a first generation sufferer. Teeth with radiographic or histologic features of DD occur in a number of disorders such as calcinosis, Ehlers-Danlos syndrome, and the brachioskeletogenital syndrome [9]. Some association has also been reported between dentine dysplasia and osseous changes in addition to sclerotic bone formation [10] but our patient had no signs of other pathologic conditions.

There were no variations in the morphology of the affected teeth in our patient but there are reports that have suggested possible variations in the morphology of teeth affected by this type of dysplasia [11,12]. Histopathologically, the peri-apical radiolucent areas seen in most cases of DD have been interpreted as radicular cysts, however, in some cases, a diagnosis of peri-apical granuloma has been reported [13].

Management of patients with dentinal dysplasia has presented dentists with many problems. Extraction has been suggested as a treatment alternative for teeth with pulp necrosis and peri-apical abscess. Follow-up and routine conservative treatment is another choice of treatment plan in DD [13]. Another approach for the treatment of teeth with DD has included peri-apical surgery and retrograde filling, which is recommended in teeth with long roots [13,14]. Since these patients usually have early exfoliation of the teeth and, consequently, maxillomandibular bony atrophy, treatment with a combination of onlay bone grafting and a sinus lift technique to accomplish implant placement can be used successfully [15].

## Conclusion

Dentin dysplasia type I is a rare inherited abnormality of the dentin that leads to premature exfoliation of the primary and permanent teeth. Early diagnosis of the condition is important for initiation of effective preventive treatment. In this regard, the pediatric dentist has an important role in the early diagnosis of this disorder and in guiding patients in the selection of measures to prolong the retention of affected teeth.

## Abbreviations

DSPP: dentin sialophosphoprotein.

## Consent

Written informed consent was obtained from the patient's parents for publication of this case report and any accompanying images. A copy of the written consent is available for review by the Editor-in-Chief of this journal.

## Competing interests

The authors declare that they have no competing interests.

## Authors' contributions

LT wrote and supervised the manuscript. FM carried out the pathologic issues and took the ground section. MM and LM carried out all dental treatments and completed the literature review.
